# Eptifibatide and abciximab inhibit insulin-induced focal adhesion formation and proliferative responses in human aortic smooth muscle cells

**DOI:** 10.1186/1475-2840-7-36

**Published:** 2008-12-23

**Authors:** Alokkumar Pathak, Renyi Zhao, Jianhua Huang, George A Stouffer

**Affiliations:** 1 Carolina Cardiovascular Biology Center, University of North Carolina, Chapel Hill, NC, USA; 2 Division of Cardiology, University of North Carolina, Chapel Hill, NC, USA

## Abstract

**Background:**

The use of abciximab (c7E3 Fab) or eptifibatide improves clinical outcomes in diabetics undergoing percutaneous coronary intervention. These β_3 _integrin inhibitors antagonize fibrinogen binding to α_IIb_β_3 _integrins on platelets and ligand binding to α_v_β_3 _integrins on vascular cells. α_v_β_3 _integrins influence responses to insulin in various cell types but effects in human aortic smooth muscle cells (HASMC) are unknown.

**Results and discussion:**

Insulin elicited a dose-dependent proliferative response in HASMC. Pretreatment with m7E3 (an anti-β_3 _integrin monoclonal antibody from which abciximab is derived), c7E3 or LM609 inhibited proliferative responses to insulin by 81%, 59% and 28%, respectively. Eptifibatide or cyclic RGD peptides completely abolished insulin-induced proliferation whereas tirofiban, which binds α_IIb_β_3 _but not α_v_β_3_, had no effect. Insulin-induced increases in c-Jun NH_2_-terminal kinase-1 (JNK1) activity were partially inhibited by m7E3 and eptifibatide whereas antagonism of α_v_β_3 _integrins had no effect on insulin-induced increases in extracellular signal-regulated kinase (ERK) activity. Insulin stimulated a rapid increase in the number of vinculin-containing focal adhesions per cell and treatment with m7E3, c7E3 or eptifibatide inhibited insulin-induced increases in focal adhesions by 100%, 74% and 73%, respectively.

**Conclusion:**

These results demonstrate that α_v_β_3 _antagonists inhibit signaling, focal adhesion formation and proliferation of insulin-treated HASMC.

## Background

Individuals with insulin resistance states and elevated levels of circulating insulin, the prototype of which is type II diabetes, are more prone to develop vascular disease and less likely to benefit from available treatments compared to non-diabetic individuals[1]. Abciximab and eptifibatide, two widely used integrin inhibitors, improve mortality in diabetics undergoing percutaneous coronary intervention (PCI). In a pooled analysis of three large clinical trials, abciximab was associated with a 44% reduction in one year mortality in diabetics (4.5% in patients receiving placebo and 2.5% in patients receiving abciximab)[2]. Similarly, eptifibatide was associated with a reduction in one year mortality in diabetics (3.5% in patients receiving placebo and 1.3% in patients receiving eptifibatide) in the Enhanced Suppression of the platelet IIb/IIIa Receptor with Integrilin Therapy (ESPRIT) trial[3].

Abciximab and eptifibatide, in addition to inhibiting platelet aggregation via antagonism of fibrinogen binding to α_IIb_β_3 _integrins, also antagonize ligand binding to α_v_β_3 _integrins on vascular cells[4,5]. Recent studies in cultured cells have revealed considerable cross-talk between α_v_β_3 _integrins and insulin receptor-mediated signals. Vuori and Ruoslahti[6] found that α_v_β_3 _integrins associate with insulin-receptor substrate-1 (IRS-1), a docking protein that phosphorylates on tyrosine following insulin-receptor activation and binds SH2 domain-containing proteins that propagate the insulin signal. Moreover, α_v_β_3 _integrins associated with tyrosine phosphorylated insulin receptors and other, as yet unidentified, tyrosine phosphorylated proteins in insulin-treated fibroblasts[7]. These associations were specific for α_v_β_3 _integrins and proliferative responses to insulin were enhanced by extracellular matrices that ligated α_v_β_3 _integrins. More recently, Lopez-Alemany et al. reported that plasminogen activator inhibitor-1 (PAI1) competes with α_v_β_3 _integrins for binding to vitronectin and by this mechanism blocks insulin-induced migration in NIH3T3 cells and human umbilical vein endothelial cells[8].

Given the important role of smooth muscle cell (SMC) proliferation in atherosclerosis progression and in revascularization failures, the present studies were performed to explore the hypothesis that abciximab and eptifibatide inhibit proliferative responses of human aortic SMC (HASMC) to insulin via antagonizing α_v_β_3 _integrins.

## Methods

### Cell culture, proliferation assays and flow cytometric analysis

HASMC were obtained from Clonetics (San Diego, CA) and maintained in culture as previously described[4]. SMC between passages 4 and 15 were used in these studies. The cells were grown in media that was a 1:1 mixture of regular DMEM and smooth muscle proliferation medium with a glucose concentration of 15.27 mM. Cell proliferation, flow activated cell sorting (FACS) analysis, apoptosis assays, focal adhesion assays and cell adhesion assays were performed as previously described[4,9].

### Reagents

m7E3 and c7E3 Fab were provided by Centocor (Malvern, Pa). Eptifibatide was provided by Cor Therapeutics (South San Francisco, CA). Insulin and peptide integrin inhibitors were purchased from Sigma (St. Louis, MO).

### Transfection and selection of stable β_3 _integrin expressing HEK cells

pcDNA-1neo constructs encoding full-length β_3 _subunits were a gift of D. Cheresh (Scripps Research Institute, La Jolla, CA) and have been previously described[10]. β_3 _integrin-deficient HEK 293 cells (ATCC; Manassas, VA) were transfected using the FuGENE Transfection Reagent (Boehringer Mannheim) and stable cell lines established as previously described[5].

### JNK1 kinase activity assay

HASMC were grown to subconfluence and then growth arrested for 48 hours in DMEM containing 0.1% FBS. Cells were pretreated with m7E3, c7E3 or eptifibatide for 1 hour, and then stimulated for 10 min at 37°C with 1 uM Insulin (Sigma). Cells were washed twice with ice-cold PBS containing 0.5 mM vanadate and then lysed with ice-cold cell lysis buffer plus protease inhibitor cocktail (Roche Diagnostics GmbH) on ice for 10 minutes. JNK1 kinase activity was measured using a GST-c-JUN pull-down assay as previously described[9].

### Statistical analysis

Results are expressed as mean ± standard deviation unless otherwise stated. One way analysis of variance followed by the Dunnett's multiple range test was used to analyze data. A p value of less than or equal to 0.05 was considered statistically significant. Triplicate wells were analyzed for each experiment and each experiment was performed independently a minimum of three times.

## Results

### HASMC express α_v_β_3 _integrins

**Figure 1:**
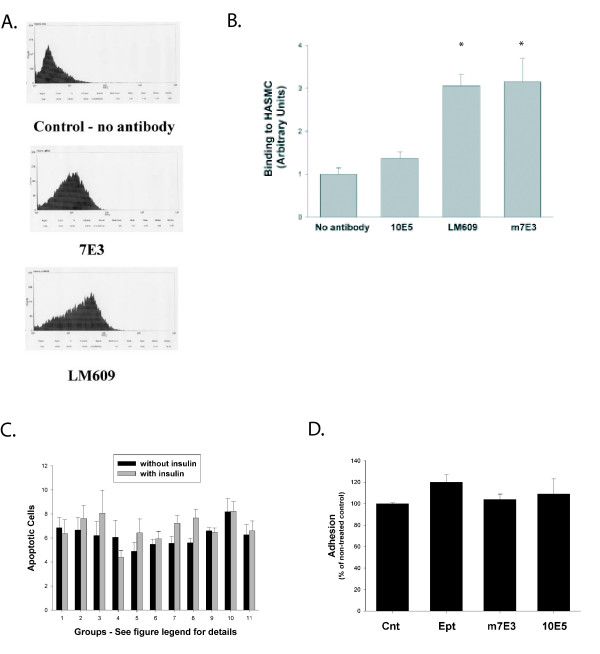
**Expression of α_v_β_3 _integrins by HASMC and effect of α_v_β_3 _antagonists on cell adhesion and apoptosis**. Results of a representative FACS experiment (A) and data from 5 independent experiments (B) are shown (* = p < 0.05 vs no antibody or vs 10E5). HASMC growth-arrested for 72 hours were treated with insulin (1 μmol/L) or vehicle ± integrin inhibitors. 48 hours later (120 hours after the addition of quiescent media), Annexin V staining was determined (C). The groups are as follows: 1 – no inhibitor, 2 – 10E5, 3 – m7E3, 4 – c7E3, 5 – LM609, 6 – eptifibatide, 7 – tirofiban, 8 – cRGD, 9 – RGD, 10 – RGE and 11 – RAD. HASMC in suspension were incubated with eptifibatide, RGE peptides, m7E3 or 10E5 and then added to non-coated tissue culture plates. Cell adhesion was determined as described in Methods (D). Concentrations of inhibitors were as follows: m7E3, c7E3, LM609 and 10E5 (30 μg/ml); eptifibatide, GRGDS, GRDGS or GRADSP peptides (10 μmol/L) and tirofiban (30 μmol/L).

Flow cytometry was performed utilizing LM609, a monoclonal antibody that binds α_v_β_3 _integrins with high specificity[11], m7E3, a monoclonal anti-β_3 _integrin antibody from which abciximab is derived, and 10E5, a monoclonal antibody that binds α_IIb_β_3 _but not α_v_β_3_. Results demonstrated that HASMC express α_v_β_3 _and that β_3 _subunits form heterodimers primarily, if not solely, with α_v _subunits in HASMC (figures 1A and 1B; binding to HASMC in arbitrary binding units: no antibody 1.0 ± 0.2, 10E5 1.4 ± 0.2, m7E3 3.1 ± 0.3 and LM609 3.2 ± 0.5; p < 0.05 for m7E3 vs. 10E5 and LM609 vs. 10E5).

Apoptosis, as determined by Annexin V binding to the cell surface, was present in less than 10% of HASMC maintained in quiescent media for 120 hours (figure 1C) and did not increase with insulin treatment. Treatment with various integrin inhibitors for 48 hours after HASMC had been maintained in quiescent media for 72 hours did not increase the number of apoptotic SMC, either with or without insulin. At the concentrations used in these studies, neither m7E3, c7E3 nor eptifibatide had any effect on cell adhesion (figure 1D). These results are consistent with prior studies showing that attachment of HASMC to non-coated tissue culture plates is primarily mediated by various β_1_-integrins[12].

### α_v_β_3 _antagonists inhibit insulin induced proliferation of HASMC

**Figure 2:**
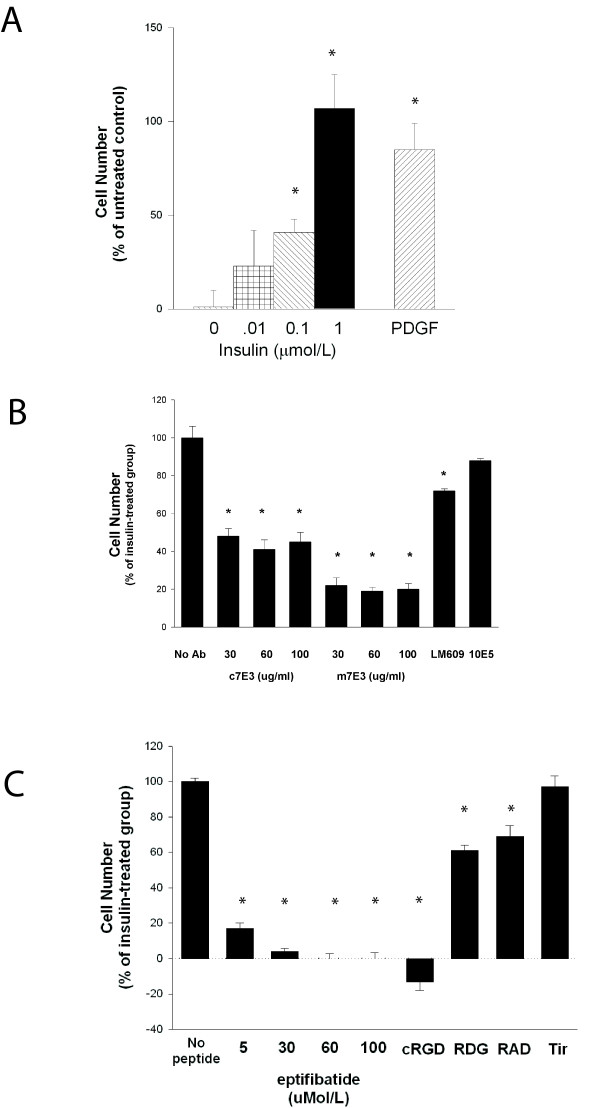
**Effect of α_v_β_3 _antagonists on insulin-induced proliferation**. Growth arrested HASMC were treated with insulin (various concentrations), PDGF-BB (1.4 nmol/L) or vehicle and cell number was determined three days later (A). In the experiments represented in panels B and C, HASMC were treated with antibodies or peptides as indicated for one hour prior to addition of insulin (1 μmol/L). Concentrations of antibodies and peptides were the same as in figure 1 unless indicated. [* = p < 0.05 vs control (A); * = p < 0.05 vs 10E5 (B); * = p < 0.05 vs tirofiban (C)]

Insulin elicited a dose-dependent proliferative response of HASMC that were growth arrested in 0.5% FBS for 72 hours prior to treatment. The proliferative response, as determined by cell number assays three days after treatment, to insulin at a concentration of 1 μmol/L (107 ± 18% increase in cell number; n = 7; range 25 – 168%) was similar to that elicited by platelet-derived growth factor-BB (PDGF-BB) (figure 2A; 85 ± 14%; n = 3; p = ns compared to insulin).

Treatment of HASMC with m7E3 (30 μg/ml) for one hour prior to the addition of insulin inhibited proliferative responses by approximately 70% (figure 2B). The inhibitory effects of 60 μg/ml or 100 μg/ml of m7E3 on insulin-induced proliferation were the same as that observed with 30 μg/ml (p = ns for differences between various concentrations).

Pretreatment with LM609 at the same dose also inhibited proliferative responses to insulin but to a lesser extent. m7E3 had a more profound inhibitory effect than LM609 even though the affinity of m7E3 for HASMC (K_D _= 3.8 ± 0.4 nmol/L) is an order of magnitude less than that of LM609 for HASMC (K_D _= 0.18 ± 0.01 nmol/L)[5]. Pretreatment with 10E5 had no effect.

c7E3 Fab (abciximab) is a chimeric antigen binding fragment derived from m7E3 which was designed to minimize antigenicity when given to humans. It contains the heavy and light chain variable regions from the murine antibody attached to the constant regions of human IgG1 and kappa chains, respectively. Pretreatment with c7E3 (30 μg/ml) inhibited 56% of the proliferative response to insulin. There was no additional inhibitory effect observed with higher concentrations of c7E3.

Two other β_3 _integrin antagonists, eptifibatide and tirofiban, are used in clinical medicine in addition to abciximab. Pretreatment of HASMC with eptifibatide at a concentration of 5 μmol/L blocked 83% of insulin-induced proliferation (figure 2C). At higher concentrations, eptifibatide completely abolished insulin-induced proliferation. In contrast, tirofiban, a nonpeptide derivative of tyrosine that does not bind α_v_β_3 _integrins, had no effect.

Cyclic RGD peptides (GPenGRGDSPCA; cRGD) are specific α_v _antagonists that recognize both α_v_β_3 _and α_v_β_5_[13]. cRGD block α_v_β_3_-mediated migration and proliferation of rat aortic SMC, α_v_β_5_-mediated adhesion of RASMC to vitronectin and neointimal formation following rat carotid artery balloon injury[9,13,14]. At a concentration of 10 μmol/L, cRGD completely inhibited insulin-induced proliferation (figure 2C). RDG and RAD peptides, used as controls, blocked 39% and 31% of the proliferative response to insulin, respectively (p < 0.05 compared to insulin alone).

### Inhibitory effects of m7E3 are mediated by α_v_β_3 _antagonism

**Figure 3:**
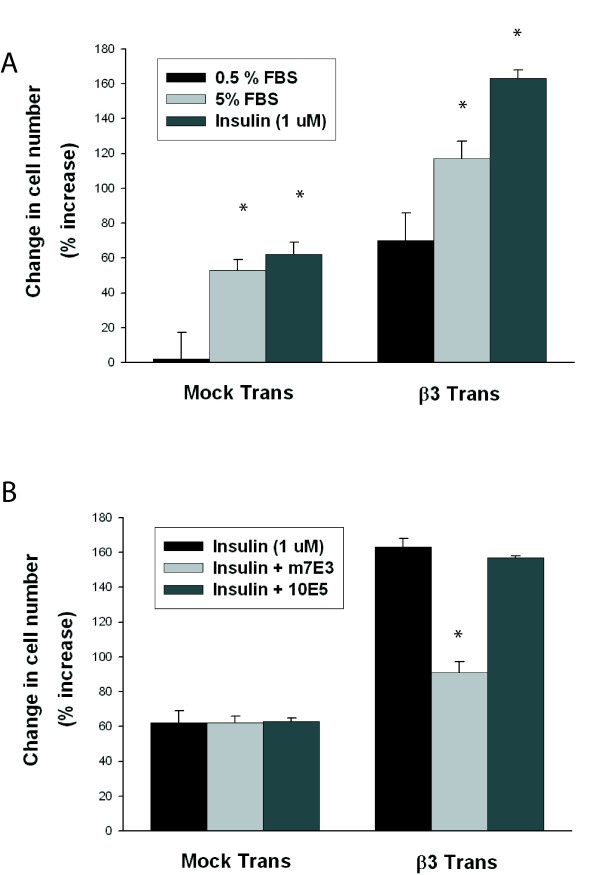
**Effects of m7E3 on proliferative responses of HEK cells**. β_3 _integrin-deficient HEK cells were transfected with an empty vector or pcDNA-1neo constructs encoding full-length β_3 _integrin subunits. The cells were grown in serum-containing media (0.5% or 5%) or insulin (1 μmol/L) for five days (A). In panel B, insulin (1 μmol/L) was added ± m7E3 or 10E5 (20 μg/ml). Cell number assays were performed 5 days later. [* – p < 0.05 vs 0.5% FBS in A and * – p < 0.05 vs insulin or insulin + 10E5 in B].

Further confirmation that the inhibitory effects of m7E3 on responses to insulin were mediated via α_v_β_3 _antagonism was provided by studies in human embryonic kidney (HEK) cells that express α_v _subunits but not β_3 _subunits. These cells were transfected with pcDNA-1neo constructs[10] encoding β_3 _integrin subunits and stable expression of β_3 _integrins and formation of α_v_β_3 _complexes was confirmed by immunoprecipitation with LM609 followed by Western analysis[5]. HEK cells that expressed β_3 _subunits proliferated at a rate that was 46% greater than mock-transfected HEK cells when grown in 5% serum on standard tissue culture plates (figure 3A). Interestingly, β_3_-HEK cells showed a significant proliferative response to 0.5% serum in contrast to mock-transfected HEK cells. Insulin was a potent mitogen for both β_3_-transfected and mock-transfected HEK cells; in both cell types proliferative responses to insulin were greater than to 5% serum. But, whereas m7E3 had no effect on insulin-induced proliferation in mock-transfected HEK cells, m7E3 inhibited approximately 60% of insulin-induced proliferation in β_3_-transfected HEK cells (figure 3B).

### α_v_β_3 _antagonists inhibit insulin-induced JNK, but not insulin-induced ERK, activity

**Figure 4:**
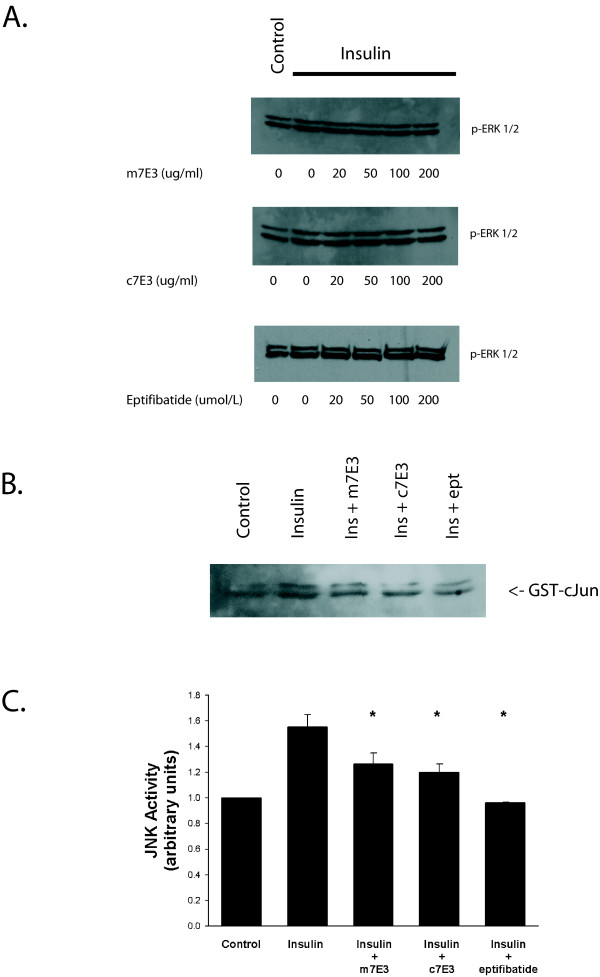
**Effect of c7E3, m7E3 and eptifibatide on insulin-induced ERK and JNK activation**. Growth arrested HASMC were treated with m7E3, c7E3 or eptifibatide (indicated concentrations in A, both antibodies were used at 30 μg/ml and eptifibatide at 10 μmol/L in B and C) as indicated for one hour. Insulin (1 μmol/L) or vehicle was added and ten minutes later ERK and JNK1 activity were determined as described in Methods.

Exposure to insulin results in the stimulation of multiple signaling pathways[15,16] but mitogen activated protein kinases (MAPK) are generally thought to regulate biological actions related to growth and proliferation[17]. Since integrins mediate activation of extracellular signal-regulated kinase (ERK)[18] and c-Jun NH_2_-terminal kinase-1 (JNK1; also known as stress-activated protein kinase-1)[9] we next sought to determine if α_v_β_3 _antagonists influenced insulin-induced ERK or JNK1 activity. Treatment with insulin elicited a mild increase in ERK 1/2 phosphorylation but pretreatment with eptifibatide, m7E3 or c7E3, at concentrations that reduced proliferation, had no effect on this response (figure 4A). In particular, neither m7E3 at a concentration that reduced insulin-induced proliferation by approximately 80%, c7E3 at a concentration that reduced insulin-induced proliferation by approximately 55% nor eptifibatide at a concentration that inhibited insulin-induced proliferation by 83% had any discernible effect on levels of tyrosine phosphorylated ERK.

Previous studies have shown JNK1 is phosphorylated in response to treatment of HASMC with insulin[19]. To determine the effect of α_v_β_3 _antagonists on insulin-induced activation of JNK1, we utilized an *in vitro *immunocomplex kinase assay with GST-c-jun as the substrate. Others have previously shown using the same experimental system that α-thrombin stimulation of JNK-1 activity is associated with subsequent increases in c-jun expression, AP-1-DNA binding activity and AP-1 transactivation activity[20]. Pretreatment with m7E3, c7E3 or eptifibatide for one hour reduced insulin-induced JNK1 activity measured 10 minutes after treatment of HASMC (figures 4B and 4C).

### Insulin stimulates the formation of focal adhesions and this effect is partially inhibited by α_v_β_3 _antagonists

**Figure 5:**
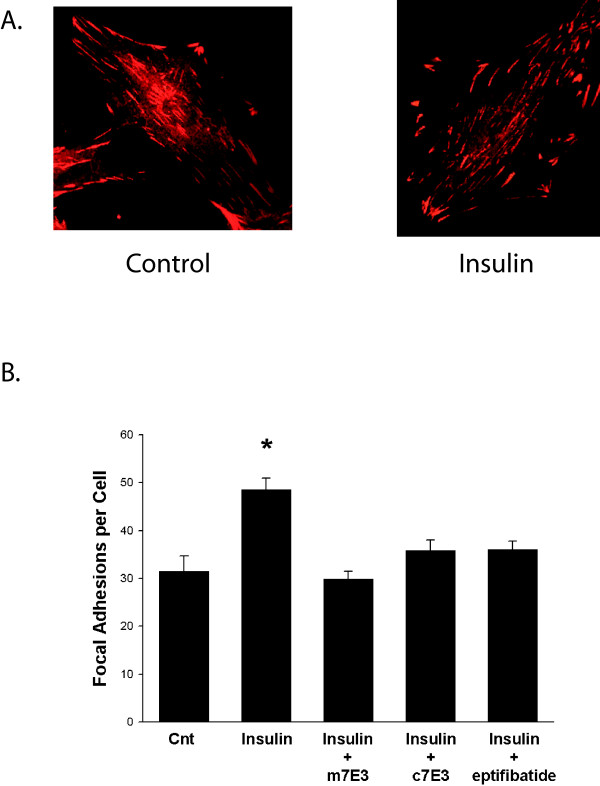
**Effect of c7E3, m7E3 and eptifibatide on insulin-induced focal adhesion formation**. Growth arrested HASMC were treated with m7E3, c7E3 (both antibodies were used at 30 μg/ml) or eptifibatide (10 μmol/L) as indicated for one hour. Insulin (1 μmol/L) or vehicle were added and five minutes later, focal adhesions in 50 representative cells were determined using vinculin staining. Data represent the mean ± SEM from four independent experiments. [* – p = 0.05 relative to insulin].

There is evidence that efficient activation of JNK1 in SMC occurs at focal adhesions, sites where integrins, cytoskeletal proteins and signaling proteins converge[21]. Focal adhesions form at the ends of F-actin stress fibers and transmit tension between the contractile apparatus and extracellular matrix. Previously we found that α_v_β_3 _antagonists inhibited JNK1 activation and focal adhesion formation in SMC in response to treatment with α-thrombin[9]. Treatment with insulin resulted in a 66% increase in focal adhesions (as determined by vinculin staining) within 5 minutes (figure 5). In a growth arrested, quiescent state there were 31 ± 3 focal adhesions per HASMC and this number increased to 49 ± 2 following five minute exposure to insulin. Pretreatment with m7E3 completely inhibited insulin-induced increases in focal adhesions. Insulin-induced increases in focal adhesions were inhibited by 74% and 73% by pretreatment with c7E3 or eptifibatide, respectively.

## Discussion

Antagonism of α_v_β_3 _integrins markedly inhibits proliferative responses of HASMC to insulin. This conclusion is based on our findings that insulin-induced proliferation was inhibited by anti-β_3 _integrin monoclonal antibody (m7E3), chimeric antigen binding fragment of 7E3 (c7E3), anti-α_v_β_3 _monoclonal antibody (LM609), anti-β_3 _peptides (eptifibatide) and anti-α_v _peptides (cRGD). These studies add to the wealth of data that α_v_β_3 _antagonists have profound effects on SMC proliferation and migration, two mechanisms that play central roles in vascular pathology. In various studies, α_v_β_3 _antagonists have been shown to inhibit proliferative responses of SMC to insulin, thrombospondin, thrombin, IGF-1, osteopontin, Del1 and transforming growth factor-β and to inhibit migratory responses of SMC to insulin-like growth factor-1 – (IGF-1), PDGF, vitronectin, thrombospondin and osteopontin (reviewed in[22]). In animal models, α_v_β_3 _antagonists have been shown to reduce SMC migration, SMC proliferation and neointima formation following vascular injury[14,23].

In this study, insulin stimulated a proliferative response in quiescent HASMC maintained in 0.5% FBS that was similar in magnitude to that observed with PDGF-BB. These results are consistent with previous studies which showed that insulin can stimulate proliferation of cultured SMC and also enhance proliferative responses to other mitogens[24-26]. Insulin has also been found to stimulate neointimal formation and proliferation of organ cultures of saphenous veins and internal mammary arteries[27]. Saphenous vein responses to insulin were similar in magnitude to those observed with PDGF-BB whereas insulin was a less efficacious mitogen than PDGF-BB in internal mammary cultures. In contrast to these studies, Obata et al.[28] found that low concentrations of insulin stimulated insulin receptor substrate-1 phosphorylation and amino acid uptake but not thymidine incorporation into DNA in rat aortic SMC. The concentrations of insulin used by Obata et al (1–10 nmol/L) were approximately 100 fold lower than used in the present studies.

We found that focal adhesions as delineated by anti-vinculin staining formed rapidly following treatment of quiescent HASMC with insulin and that α_v_β_3 _antagonists partially inhibited this response. Vinculin is one of the first actin binding proteins recruited into focal adhesions[29]. It is widely used as a marker of 'classic' focal adhesions, which are usually located at the cell periphery, are highly tyrosine phosphorylated, and contain α_v_β_3 _integrins. Vinculin is also found in focal complexes, which are short-lived structures that mature into focal adhesions.

Recent studies have highlighted the existence of two major signaling pathways that are initiated by insulin binding to the insulin receptor and which mediate insulin action[15,16]. One pathway, which involves IRS proteins and phosphatidylinositol 3-kinase, appears to be responsible for most, if not all, of the metabolic aspects of insulin action. The second signaling pathway, involving Ras and mitogen activated protein kinase (MAPK), is responsible for proliferative responses to insulin. Doronzo et al reported that insulin increased ERK 1/2 and JNK1 phosphorylation in HASMC in a time dependent manner beginning within 5 minutes of treatment[19]. In the present studies, we found that antagonism of α_v_β_3 _inhibited insulin-induced activation of JNK1, but not ERK 1/2, in HASMC. JNK1 is a member of the mitogen activated protein kinase superfamily that is activated by dual phosphorylation at a Thr-Pro-Tyr motif and once activated, functions to phosphorylate c-jun at amino terminal serine regulatory sites which increases activity of the transcription factor AP-1. A clear link between JNK1 activation and proliferation in insulin-treated HASMC has not been established although JNK antagonists has been reported to inhibit proliferation of cultured SMC in other systems[30,31]. In studies utilizing the rat carotid balloon injury model, transfection of a dominant negative JNK prior to injury prevented neointimal formation and markedly suppressed SMC proliferation in both the intima and the media after rat carotid artery injury[32].

The inhibitory effect of α_v_β_3 _antagonists on JNK1 activation occurs in response to a variety of stimuli. In addition to inhibiting JNK1 activation in response to insulin, α_v_β_3 _antagonists inhibit α-thrombin-induced[9] and TFB-induced[33] JNK1 activation in SMC. Several potential mechanisms that might explain these results are suggested by recent studies. Activation of the small G protein Rho is mediated by integrin engagement[34] and recently Ohtsu et al reported that activation of Rho, and its effector Rho-kinase/ROCK was required for angiotensin II-induced JNK activation in SMC. Alternatively, studies in myocytes[35] and HEK cells[21,36] have implicated the formation of protein complexes involving focal adhesion kinase in activation of JNK.

α_v_β_3 _antagonists inhibited insulin-induced proliferation without blocking ERK 1/2 phosphorylation. The biochemical steps involved in signal transduction through the ERK pathway are well established but less is known about how these signals are implemented into specific biological responses, and in particular the role of intracellular localization of members of this pathway. Following activation, ERK localizes to different subcellular compartments, including focal adhesions, and phosphorylates specific proteins leading to cellular responses. The specificity of the biological response is likely to be at least partially controlled by the localization of signaling, which enables ERK activity to be directed towards specific targets. Integrin engagement is necessary for active ERK localization to focal adhesions suggesting that a potential mechanism whereby integrin antagonism could inhibit growth but not ERK phosphorylation is via interrupting ERK targeting[37].

Tirofiban had no effect on insulin-induced proliferation, consistent with prior studies showing that tirofiban does not antagonize α_v_β_3_[5]. The different affinities of eptifibatide and tirofiban for α_v_β_3 _are not surprising given that eptifibatide is a synthetic, cyclic peptide with a Lys-Gly-Asp (KGD) sequence whereas tirofiban is a nonpeptide derivative of tyrosine. Tirofiban was used at a concentration of 30 μmol/L and eptifibatide was used at various concentrations between 5 and 100 μmol/L in our studies. This concentration of tirofiban is approximately 700 fold greater than peak plasma concentration observed in patients (40 ng/ml) receiving a continuous infusion[38]. There is wide inter-individual variation in plasma levels however, and it is also unknown whether tirofiban concentrations are higher within the vessel wall than in plasma.

Intimal thickening due to abnormal proliferation of vascular smooth muscle cells is the major cause of revascularization failures in diabetics. Since α_v_β_3 _integrin expression is upregulated in atherosclerotic lesions and at sites of balloon angioplasty, these results suggest that one way to potentially regulate insulin effects on SMC at sites of vascular healing is via antagonism of α_v_β_3_.

## Competing interests

The authors declare that they have no competing interests.

## Authors' contributions

AP performed the flow cytometry, proliferation and binding assays. He also assisted in interpreting the data and writing the manuscript. RZ performed the focal adhesion assays and immunoflouresence studies. JH performed the western blots and JNK assays. GAS conceived of the study, participated in the design, coordinated the interpretation of the data and drafted the manuscript. All authors read and approved the final manuscript.
